# Paravalvular Regurgitation: Clinical Outcomes in Surgical and Percutaneous Treatments

**DOI:** 10.5935/abc.20160086

**Published:** 2016-07

**Authors:** Carlos Passos Pinheiro, Daniele Rezek, Eduardo Paiva Costa, Edvagner Sergio Leite de Carvalho, Freddy Antonio Brito Moscoso, Percy Richard Chavez Taborga, Andreia Dias Jeronimo, Alexandre Antonio Cunha Abizaid, Auristela Isabel de Oliveira Ramos

**Affiliations:** Instituto Dante Pazzanese de Cardiologia, São Paulo, SP - Brazil

**Keywords:** Heart Valve Diseases / surgery, Aortic Valve Insufficiency / surgery, Mitral Valve Insufficiency / surgery, Heart Valve Prosthesis, Echocardiography, Transesophageal

## Abstract

**Background:**

Paravalvular regurgitation (paravalvular leak) is a serious and rare complication associated with valve replacement surgery. Studies have shown a 3% to 6% incidence of paravalvular regurgitation with hemodynamic repercussion. Few studies have compared surgical and percutaneous approaches for repair.

**Objectives:**

To compare the surgical and percutaneous approaches for paravalvular regurgitation repair regarding clinical outcomes during hospitalization and one year after the procedure.

**Methods:**

This is a retrospective, descriptive and observational study that included 35 patients with paravalvular leak, requiring repair, and followed up at the Dante Pazzanese Institute of Cardiology between January 2011 and December 2013. Patients were divided into groups according to the established treatment and followed up for 1 year after the procedure.

**Results:**

The group submitted to percutaneous treatment was considered to be at higher risk for complications because of the older age of patients, higher prevalence of diabetes, greater number of previous valve surgeries and lower mean creatinine clearance value. During hospitalization, both groups had a large number of complications (74.3% of cases), with no statistical difference in the analyzed outcomes. After 1 year, the percutaneous group had a greater number of re-interventions (8.7% vs 20%, p = 0.57) and a higher mortality rate (0% vs. 20%, p = 0.08). A high incidence of residual mitral leak was observed after the percutaneous procedure (8.7% vs. 50%, p = 0.08).

**Conclusion:**

Surgery is the treatment of choice for paravalvular regurgitation. The percutaneous approach can be an alternative for patients at high surgical risk.

## Introduction

Paravalvular regurgitation or leak is a severe and uncommon complication associated with heart valvular prosthesis implantation. It consists in an abnormal communication between the implanted prosthesis structure and the cardiac tissue, generating a turbulent blood flow with varied clinical consequences.^1^

Studies with transesophageal echocardiography (TEE) after heart valve replacement surgery have reported an incidence of paravalvular leak with hemodynamic repercussion ranging from 3% to 6%, and with no hemodynamic repercussion of 20%,^2-4^ more often found with mechanical mitral valve prosthesis.^5-8^

Major determinants of paravalvular leak are as follows: calcification or fragility of the valvular ring; infective endocarditis (IE); technical difficulties associated with suturing; prosthetic size and shape; previous mitral valve regurgitation; acute myocardial infarction; and Marfan's syndrome.^9^

The leak can appear early when related to surgical technical aspects, or late, caused by suture dehiscence or infection.^10^ Clinical presentation varies. Around 5% of patients are symptomatic, manifesting signs and symptoms of heart failure (HF), hemolytic anemia or IE.^11-12^

Transthoracic echocardiography (TTE) often does not allow differentiating between periprosthetic leak and regurgitation secondary to degenerative changes of bioprostheses. Three-dimensional (3D) TEE is the most indicated method to assess in details the position, severity and hemodynamic repercussion.^13-14^ In addition, 3D TEE contributes to the strategic planning of percutaneous closure or of surgical treatment.^15-18^

Clinical treatment of paravalvular regurgitation is mainly a palliative measure to control symptoms.^19-21^ Surgery is the procedure of choice in the presence of IE, significant dysfunction or mechanic instability of the prosthesis. However, the significant increase in mortality with subsequent surgeries is well known. When the indication for the new intervention is due to paravalvular leak, mortality of the first reoperation reaches 30%.^22-23^ Currently, for patients with prohibitive surgical risk or multiple previous surgeries, percutaneous closure with implantation of occlusion devices appears as a strategy of feasible, promising and less invasive treatment.

Therefore, exploring that theme is important considering the small number of studies comparing between surgical and percutaneous approaches of paravalvular leaks and their long-term results.

This study was aimed at comparing the surgical and percutaneous approaches to correct paravalvular regurgitation regarding clinical outcome during hospitalization and 1 year after the procedure.

## Methods

This is a retrospective, descriptive, observational study including patients diagnosed with paravalvular regurgitation with clinical consequences (HF, hemolysis or IE), requiring repair (surgical or percutaneous), followed-up at the Dante Pazzanese Institute of Cardiology from January 2011 to December 2013.

During that period, 35 patients diagnosed with paravalvular leak and requiring repair were assessed at the Valvulopathy Medical Unit. All diagnoses were confirmed with TEE. The decision about the type of approach (surgical or percutaneous) was based on the patient's surgical risk, considering age, number of previous surgeries and associated comorbidities. The Valve Clinic of our hospital was responsible for the decision, and later the cases considered at high surgical risk were referred to the Heart Team, aiming at percutaneous closure of the leak. Patients with paravalvular defects affecting at least one third of the circumference of the prosthetic ring were considered ineligible for the percutaneous procedure.

The percutaneous repair was performed under general anesthesia and guided by TEE and radioscopy. Mitral valve leak closure was performed via anterograde approach (access via femoral vein and transeptal puncture), retrograde approach (access via femoral artery) or transapical approach (direct puncture), depending on the patient's anatomy and defect location. Aortic regurgitation repair was performed via retrograde approach (through the aorta). In all cases, Amplatzer Vascular Plug III (AVP III) was the closure device used. Surgical repair was performed based on the surgical techniques described in the literature.^28^

Epidemiological and clinical data and risk factors possibly involved in the pathophysiology of the leak were assessed in the entire study population. Later, the patients were divided according to the treatment established (surgical or percutaneous), aiming at comparing clinical outcomes.

Both groups were compared regarding in-hospital outcome and outcome in the first year after the procedure. The in-hospital outcomes were: death, cardiovascular complications (cardiogenic shock, HF, stroke, arrhythmia, bleeding, reoperation), non-cardiac complications (acute renal failure - ARF and infection) and hospital length of stay in days. Bleeding was defined as the need for transfusion of at least two erythrocyte concentrates. Persistent creatinine increase higher than 0.5 mg/dL was considered ARF. Infection was considered based on clinical and laboratory parameters, and the sites reported were lungs, surgical wound, urine and blood stream. The following late outcomes (1 year after the procedure) were assessed: death, re-hospitalization, reoperation, and clinical and echocardiographic data.

Data underwent statistical analysis. The results were considered statistically significant for p values below 0.05. Qualitative variables were assessed by using Fisher and chi-square tests, while quantitative variables were assessed by using non-parametric Mann-Whitney test. Survival in the first year after the repair procedure was analyzed by using Kaplan-Meier method and the *log-rank* test to compare between groups. All statistical analyses were performed with the SPSS software (SPSS™ 13.0 for Windows™ , SPSS Inc., Chicago, IL, USA).

This study project was approved by the Research Ethics Committee of the Dante Pazzanese Institute of Cardiology.

## Results

### Population characteristics

The mean age of the 35 patients submitted to the intervention was 54 ± 14 years, and 71.4% of them were of the male sex (Table 1). The highest incidence of regurgitation occurred with mitral prostheses (60% of the cases) and bioprostheses (51.4%). According to echocardiographic analysis, 60% of the leaks were classified as severe, 28.6% as moderate, and 11.4% as mild (Table 3).

**Table 1:** Patients’ characteristics

**Variables**	**Surgical group n = 25 (71.4%)**	**Percutaneous group n = 10 (28.6%)**	**Total n = 35**	**p**
Age	50 ± 13	63 ± 13	54 ± 14	0.011
**Sex**				
Male	17 (68%)	8 (80%)	25 (71.4%)	0.68
Female	8 (32%)	2 (20%)	10 (28.6%)	
**Antecedents**				
BMI	26.8 ± 6	23.1 ± 7	25 ± 5	0.13
Arterial hypertension	16 (64%)	7 (70%)	23 (65.7%)	0.99
Diabetes mellitus	0 (0%)	3 (30%)	3 (8.6%)	0.018
Dyslipidemia	10 (40%)	3 (30%)	13 (37.1%)	0.70
Stroke	3 (12%)	1 (10%)	4 (11.4%)	0.99
EF < 50%	6 (24%)	2 (20%)	8 (22.9%)	0.99
AF/Flutter	11 (44%)	3 (30%)	14 (40%)	0.70
Creatinine clearance	90 ± 38	72 ± 38	85 ± 39	0.070
Previous valve surgeries	1.72 ± 0.7	2.6 ± 1	1.97 ± 0.92	0.041

BMI: body mass index; EF: ejection fraction; AF: atrial fibrillation.

**Table 3:** Echocardiographic data

**Variables**	**Surgical group n = 25 (71.4%)**	**Percutaneous group n = 10 (28.6%)**	**Total n = 35**	**p**
**Echocardiographic measures**				
LA	53 ± 14	54 ± 14	53 ± 13	0.81
LVDD	60 ± 10	62 ± 9	60 ± 10	0.70
LVSD	41 ± 11	39 ± 11	41 ± 11	0.67
EF	58 ± 13	59 ± 16	58 ± 14	0.41
PASP	56 ± 17	73 ± 13	61 ± 17	0.013
**Leak**				0.46
Mitral	13 (52%)	8 (80%)	21 (60%)	
Aortic	11 (44%)	2 (20%)	13 (37%)	
Mitral+Aortic	1 (4%)	0 (0%)	1 (3%)	
**Prosthesis type**				0.14
Mechanical	10 (40%)	7 (70%)	17 (48.6%)	
Biological	15 (60%)	3 (30%)	18 (51.4%)	
**Leak severity**				0.39
Mild	4 (16%)	0 (0%)	4 (11.4%)	
Moderate	7 (28%)	3 (30%)	10 (28.6%)	
Severe	14 (56%)	7 (70%)	21 (60%)	

LA: left atrial diameter; LVDD: left ventricular diastolic diameter; LVSD: left ventricular systolic diameter; EF: ejection fraction; PASP: pulmonary artery systolic pressure.

Moderate to severe preoperative mitral regurgitation was diagnosed in 31.4% of the cases, followed by valve ring calcification in 25.7%. Previous IE was found in 14.3% of the patients, and acute myocardial infarction, in 8.6%. No patient had Marfan's syndrome or previous myocardial revascularization.

Based on the type of treatment chosen, the groups were as follows: percutaneous group, 10 patients (28.6%), and surgical group (valve replacement surgery), 25 patients (71.4%). In the percutaneous treatment group, patients were older (mean age of 63 ± 13 years versus (vs.) 50 ± 13 years), with statistical significance (p = 0.011).

Both groups were similar regarding the following characteristics: history of systemic arterial hypertension (64% vs. 70%, p = 0.99); dyslipidemia (40% vs. 30%, p = 0.70); preoperative stroke (12% vs. 10%, p = 0.99); ejection fraction (EF) lower than 50% (24% vs. 20%, p = 0.99); and atrial fibrillation or flutter (44% vs. 30%, p = 0.70). Mean creatinine clearance (mL/min) was 90 ± 38 in the surgical group, and 72 ± 38 in the percutaneous group (p = 0.07). The only three patients who had diabetes, belonged in the percutaneous group (8.6%, p = 0.018) (Table 1). The number of previous valve surgeries was higher in the percutaneous group (1.7 ± 0.7 vs. 2.6 ± 1), with statistically significant difference (p = 0.041).

### Clinical presentation at the time of diagnosis

Most patients presented with dyspnea and New York Heart Association (NYHA) functional class (FC) III (FC I: 25.7%, FC II: 25.7%, FC III: 40%, FC IV: 8.6%). Clinical and laboratory signs of hemolysis were evident in 42% of patients [32% of the surgical group, and 70% of the percutaneous group (p = 0.04)] (Table 2). Only two patients were diagnosed with IE or abscess, and belonged in the surgical group (8% vs. 0%, p = 0.99).

**Table 2:** Clinical and laboratory findings at presentation

**Variables**	**Surgical group n = 25 (71.4%)**	**Percutaneous group n = 10 (28.6%)**	**Total n = 35**	**p**
**HF**				0.60
FC I	8 (32%)	1 (10%)	9 (25.7%)	
FC II	6 (24%)	3 (30%)	9 (25.7%)	
FC III	9 (36%)	5 (50%)	14 (40%)	
FC IV	2 (8%)	1 (10%)	3 (8.6%)	
Hemolysis	8 (32%)	7 (70%)	15 (42%)	0.04
IE / Abscess	2 (8%)	0 (0%)	2 (5.7%)	0.99
**Laboratory data**				
Hemoglobin	12 ± 2	9.7 ± 1.7	11.44 ± 2.36	0.01
LDH	1327 ± 1665	1286 ± 849	1314 ± 1435	0.40
IB	0.81 ± 0.68	1.07 ± 0.99	0.89 ± 0.78	0.39
Creatinine	1.23 ± 1.46	1.11 ± 0.41	1.19 ± 1.25	0.30
BNP	12566 ± 17872	1694 ± 1412	7130 ± 12807	0.40

HF: heart failure; FC: functional class; IE: infective endocarditis; LDH: lactic dehydrogenase; IB: indirect bilirubin; BNP: brain natriuretic peptide.

Regarding laboratory data, both groups had hemolytic anemia, characterized by reduced hemoglobin and high bilirubin and lactic dehydrogenase. The percutaneous group had more severe anemia (mean hemoglobin of 9.7 ± 1.7 vs. 12 ± 2, p = 0.010) (Table 2).

No difference was observed between groups regarding left atrial diameter (53 ± 14 vs. 54 ± 14 mm, p = 0.81) and left ventricular diastolic diameter (60 ± 10 vs. 62 ± 9 mm, p = 0.70), systolic diameter (41 ± 11 vs. 39 ± 11 mm, p = 0.67) and EF (58% ± 13 vs. 59% ± 16, p = 0.41). The percutaneous group had the highest mean pulmonary artery systolic pressure (56 ± 17 vs. 73 ± 13 mm Hg, p = 0.013) (Table 3).

Considering the leaks identified in the percutaneous group, 70% affected mechanical prostheses, 80% affected mitral prostheses, and, on echocardiographic assessment, 70% were classified as severe leak, while 30%, as moderate leak.

### In-hospital outcome

During hospitalization, both groups had several complications. No statistical difference was observed between them regarding the following composite outcomes: in-hospital complications (surgical vs. percutaneous group, 72% vs. 80%) and cardiac (52% vs. 50%) and non-cardiac (44% vs. 70%) complications. Two patients in the surgical group died, but none in the percutaneous group (8% vs. 0%, p = 0.99). The mean hospital length of stay was 31 days (30 ± 23 vs. 32 ± 34, p = 0.84). Comparing to previous laboratory tests, both groups showed a reduction in hemolysis during hospitalization after the procedure (reduction of 62% vs. 29%).

Table 4 shows in-hospital complications in both groups. Decompensated HF was more often in the percutaneous group (30% vs. 4%, p = 0.061).

**Table 4:** In-hospital outcome

**Variables**	**Surgical group n = 25 (71,4%)**	**Percutaneous group n = 10 (28,6%)**	**Total n = 35**	**p**
In-hospital complications	18 (72%)	8 (80%)	26 (74.3%)	0.99
Death	2 (8%)	0 (0%)	2 (5.1%)	0.99
Cardiac complications	13 (52%)	5 (50%)	18 (51.4%)	0.99
Cardiogenic shock	2 (8%)	3 (30%)	5 (14.3%)	0.12
HF	1 (4%)	3 (30%)	4 (11.4%)	0.061
Arrhythmia	12 (48%)	3 (30%)	15 (42.9%)	0.45
Stroke	1 (4%)	0 (0%)	1 (2.9%)	0.99
Bleeding	12 (48%)	3 (30%)	15 (42.9%)	0.45
Valvular reoperation	1 (4%)	2 (20%)	3 (8.6%)	0.56
Non-cardiac complications	11 (44%)	7 (70%)	18 (51.4%)	0.26
ARF	6 (24%)	3 (30%)	9 (25.7%)	0.69
Infection	9 (36%)	1 (10%)	10 (28.6%)	0.21
Hospital length of stay (days)	30 ± 23	32 ± 34	31.06 ± 26.57	0.84
Post-procedure hemolysis	3 (12%)	5 (50%)	8 (22.9%)	0.04

HF: heart failure; ARF: acute renal failure.

### Outcome 1 year after the procedure

**Figure 1:**
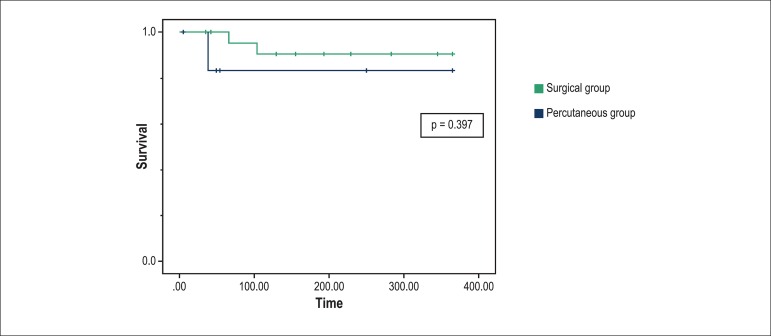
Survival curve of patients undergoing regurgitation repair in the first year after the procedure.

After a 1-year follow-up, the FC improved, 70.4% of the patients being in FC I. Percutaneous group patients required more reoperations (8.7% vs. 20%, p = 0.57) due to HF and prosthetic dysfunction. Two percutaneous group patients died. The 1-year survival curve was lower in the percutaneous group (Figure 1) (p = 0.397).

Approximately 40% of all patients had residual paravalvular leak 1 year after the procedure. In the percutaneous group, 60% of the patients remained with residual leak as compared to 30% of the surgical group patients (Table 5). However, most post-intervention leaks (46%) were considered mild and none had clinical consequences.

**Table 5:** Outcome 1 year after the procedure

**Variables**	**Surgical group n = 23 (69.7%)**	**Percutaneous group n = 10 (30.3%)**	**Total n = 33**	**p**
**HF**				0.30
FC I	16 (72.7%)	3 (60%)	19 (70.4%)	
FC II	3 (13.6%)	0 (0%)	3 (11.1%)	
FC III	2 (9.1%)	2 (40%)	4 (13.8%)	
FC IV	1 (4.5%)	0 (0%)	1 (3.7%)	
Re-hospitalization	3 (13%)	2 (20%)	5 (15.2%)	0.63
Reoperation	2 (8.7%)	2 (20%)	4 (12.1%)	0.57
Death	0 (0%)	2 (20%)	2 (6.1%)	0.08
**Echocardiographic measures**				
LA	51 ± 14	56 ± 20	51 ± 14	0.74
LVDD	58 ± 10	59 ± 10	58 ± 10	0.55
LVSD	37 ± 8.6	40 ± 12	37 ± 8.6	0.49
EF	55 ± 13	55 ± 14	55 ± 13	0.91
PASP	52 ± 20	66.5 ± 16	52 ± 20	0.11
Residual regurgitation	7 (30.4%)	6 (60%)	13 (39.9%)	0.14
Mitral	2 (8.7%)	5 (50%)	7 (53.8%)	0.08
Aortic	4 (17.3%)	1 (10%)	5 (38.5%)	0.99
Mitral-aortic	1 (4.4%)	0 (0%)	1 (7.7%)	0.99
**Leak severity**				
Mild	3 (13%)	3 (30%)	6 (18.2%)	0.34
Moderate	2 (8.7%)	1 (10%)	3 (9.1%)	0.99
Severe	2 (8.7%)	2 (20%)	4 (12.1%)	0.57

HF: heart failure; FC: functional class; LA: left atrial diameter; LVDD: left ventricular diastolic diameter; LVSD: left ventricular systolic diameter; EF: ejection fraction; PASP: pulmonary artery systolic pressure.

It is worth noting the high incidence of residual mitral regurgitation after the percutaneous procedure as compared to the surgical one, although with no statistical significance (8.7% vs. 50%, p = 0.08).

## Discussion

Paravalvular regurgitation with clinical consequences is an uncommon complication, estimated to affect 3% to 6% of patients undergoing valve replacement.^4-7^ Epidemiological records on the theme are scarce.

In this study, the diagnosis of periprosthetic leak was confirmed by use of TEE, the most appropriate method available to assess the position, severity and hemodynamic repercussion of the defect, and useful to strategically plan the repair.^13-18^

In this study, we observed a greater incidence of mitral leak, corresponding to 60% of the cases, similar to the 68% reported by Akins et al.^24^ Regarding the prosthesis type, we found 51.4% of leaks in biological valve prostheses, differently from that reported by Jindani et al.^8^ (62.5% of leaks in mechanical valve prostheses). Nevertheless, among our patients the prevalence of bioprosthesis is higher, which could justify our finding.

The major symptom was HF due to volume overload, present in 74% of the patients, with prevalence of dyspnea and FC III in 40% of the cases. Ruiz et al.^12^ have found similar results, reporting the presence of HF in 90% of their patients, most with FC III. Similarly, Genoni et al.^23^ have reported 38% of their sample with dyspnea and FC III or IV at the time of paravalvular regurgitation diagnosis. It is worth noting that 11% of the leaks, although mild, could cause symptoms, requiring intervention. The increase in BNP (mean value of 7130) reflects the cardiovascular decompensation state, being directly related to regurgitation volume, left chamber dilation and ventricular dysfunction.

Until recently, surgery was the only therapeutic option available, despite the high mortality associated with reoperation.^9,10^ Regurgitation closure by use of hemodynamic procedure has gained importance in recent years.^20,21^ When well-succeeded, clinical results are reasonable, without the significant operative mortality rates associated with reoperation. It is indicated as an alternative therapy for symptomatic patients at high risk for surgery.^3^ This study patients treated percutaneously were more critically ill, the reason why they were at higher risk for complications.

The in-hospital outcome confirmed the high morbidity and mortality of both treatment types. There was a high number of cardiac (51.4%) and non-cardiac (51.4%) complications, with no statistical difference between the groups (Table 4). An in-hospital mortality of 5.1% was found, all in-hospital deaths occurring in the surgical group. That figure differs from the high post-operative mortality of 12% reported by Genoni et al.^23^ The mean hospital length of stay of 31 days reflects the critically ill population of this study.

After 1-year follow-up, patients' symptoms improved, 70.4% of them being in FC I. High morbidity (15.2% were re-hospitalized and 12.1% underwent new re-intervention) and 6.1% mortality rate in the first year were observed, predominating in the percutaneous group.

The mortality behavior suggests that the deaths in the surgical group are related to reoperation-related complications. In the percutaneous group, the deaths occurred after hospital discharge, and can be related to the severity of the patients' illness. The survival curves in Figure 1 show no statistical difference in the first year of follow-up; however, a trend towards higher mortality is observed among the patients treated with the percutaneous technique, probably because they were more critically ill.

 On routine echocardiography one year after intervention, residual leak was identified in approximately 40% of the cases. According to the literature, the failure rate of both therapeutic approaches ranges between 12% and 35%.^23^ Leak recurrence is uncommon, as long as the underlying pathological process remains unchanged.

The present study had limitations, such as the small number of patients in each group, and the different clinical characteristics of the groups. In addition, the small sample size hindered the analysis of the diverse clinical situations involved in the paravalvular regurgitation diagnosis, such as hemolysis, HF and IE. The objective of this study was to show the characteristics of the groups and their evolution after the procedure. Our results allow us to point trends, raise hypotheses and suggest complementary studies on the subject.

## Conclusion

Paravalvular regurgitation is rare and affects mainly mitral and biological prostheses. Surgical intervention is the current treatment of choice. In the present study, the strategy of percutaneous closure of the leak, still an initial experience, was effective to improve HF and hemolysis in the sample assessed. We believe that refining the percutaneous technique will allow its indication for patients at higher risk for surgery.
